# Association of BCG Vaccine Treatment With Death and Dementia in Patients With Non–Muscle-Invasive Bladder Cancer

**DOI:** 10.1001/jamanetworkopen.2023.14336

**Published:** 2023-05-19

**Authors:** Marc S. Weinberg, Affan Zafar, Colin Magdamo, Sun Young Chung, Wesley H. Chou, Madhur Nayan, Mayuresh Deodhar, Daniel M. Frendl, Adam S. Feldman, Denise L. Faustman, Steven E. Arnold, Bella Vakulenko-Lagun, Sudeshna Das

**Affiliations:** 1 Department of Psychiatry, Massachusetts General Hospital, Boston; 2 Department of Neurology, Massachusetts General Hospital, Boston; 3 Harvard Medical School, Boston, Massachusetts; 4 Department of Urology, Massachusetts General Hospital, Boston; 5 Division of Urology, Brigham and Women’s Hospital, Boston, Massachusetts; 6 Harvard Medical School, Boston, Massachusetts; 7 Department of Urology, Oregon Health and Science University, Portland; 8 Department of Urology, New York University, New York; 9 Department of Urology, Mayo Clinic, Phoenix, Arizona; 10 Immunobiology Laboratories, Massachusetts General Hospital, Boston; 11 Department of Statistics, University of Haifa, Mt Carmel, Haifa, Israel

## Abstract

**Importance:**

The BCG vaccine—used worldwide to prevent tuberculosis—confers multiple nonspecific beneficial effects, and intravesical BCG vaccine is currently the recommended treatment for non–muscle-invasive bladder cancer (NMIBC). Moreover, BCG vaccine has been hypothesized to reduce the risk of Alzheimer disease and related dementias (ADRD), but previous studies have been limited by sample size, study design, or analyses.

**Objective:**

To evaluate whether intravesical BCG vaccine exposure is associated with a decreased incidence of ADRD in a cohort of patients with NMIBC while accounting for death as a competing event.

**Design, Setting, and Participants:**

This cohort study was performed in patients aged 50 years or older initially diagnosed with NMIBC between May 28, 1987, and May 6, 2021, treated within the Mass General Brigham health care system. The study included a 15-year follow-up of individuals (BCG vaccine treated or controls) whose condition did not clinically progress to muscle-invasive cancer within 8 weeks and did not have an ADRD diagnosis within the first year after the NMIBC diagnosis. Data analysis was conducted from April 18, 2021, to March 28, 2023.

**Main Outcomes and Measures:**

The main outcome was time to ADRD onset identified using diagnosis codes and medications. Cause-specific hazard ratios (HRs) were estimated using Cox proportional hazards regression after adjusting for confounders (age, sex, and Charlson Comorbidity Index) using inverse probability scores weighting.

**Results:**

In this cohort study including 6467 individuals initially diagnosed with NMIBC between 1987 and 2021, 3388 patients underwent BCG vaccine treatment (mean [SD] age, 69.89 [9.28] years; 2605 [76.9%] men) and 3079 served as controls (mean [SD] age, 70.73 [10.00] years; 2176 [70.7%] men). Treatment with BCG vaccine was associated with a lower rate of ADRD (HR, 0.80; 95% CI, 0.69-0.99), with an even lower rate of ADRD in patients aged 70 years or older at the time of BCG vaccine treatment (HR, 0.74; 95% CI, 0.60-0.91). In competing risks analysis, BCG vaccine was associated with a lower risk of ADRD (5-year risk difference, −0.011; 95% CI, −0.019 to −0.003) and a decreased risk of death in patients without an earlier diagnosis of ADRD (5-year risk difference, −0.056; 95% CI, −0.075 to −0.037).

**Conclusions and Relevance:**

In this study, BCG vaccine was associated with a significantly lower rate and risk of ADRD in a cohort of patients with bladder cancer when accounting for death as a competing event. However, the risk differences varied with time.

## Key Points

**Question:**

Does the BCG vaccine have a protective association with the risk of Alzheimer disease and related dementias (ADRD)?

**Findings:**

In this cohort study of 6467 patients with non–muscle-invasive bladder cancer, treatment with intravesical BCG vaccine was associated with a reduced risk of ADRD in the presence of death as a competing risk. However, the risk differences varied with time.

**Meaning:**

The findings of this study suggest that bladder cancer treatment with BCG vaccine was associated with decreased mortality, and decreased ADRD, independently; clinical trials are required to study its efficacy beyond treatment in patients with bladder cancer.

## Teaser

This cohort study evaluates the use of BCG vaccine in patients with non–muscle-invasive bladder cancer who were at risk for Alzheimer disease and related dementias.

## Introduction

Alzheimer disease and related dementias (ADRD) are progressive neurologic disorders of older adults, marked by loss of cognition, resulting in loss of independence, significant comorbidities, and death.^1^ The worldwide burden of ADRD will increase in the coming decades.^2^ While amyloid-targeting monoclonal antibody–based treatments^3^ are receiving or in line for US Food and Drug Administration approval for near-term medical use, global inaccessibility, poor efficacy, and lack of cost-effectiveness^4^ leave gaps in foreseeable treatment options for most people. Population-based prevention and treatment will likely prove the most meaningful and equitable means of solving the complex challenges of ADRD.^5^ Vaccines exemplify cost-effective population health solutions.

The immune system actively supports and protects the brain in early Alzheimer disease, but it can become functionally dysregulated—inadequate and/or overactive—later in the disease.^6^ While harnessing the immune system has revolutionized medicine in recent decades, efforts to translate genetic, epidemiologic, and biomarker-based links of immune dysregulation to ADRD treatment has been broadly unsuccessful.^7^ Several common vaccines have been associated with decreased risk of ADRD.^8^ Although most vaccines studied in the context of ADRD prevention are built into current public health recommendations for older adults,^9^ one vaccine remains an exception: BCG, a century-old live-attenuated vaccine against tuberculosis^10^ given to infants worldwide (although inconsistently across nations). The BCG vaccine is associated with numerous nonspecific beneficial effects, including reduction of all-cause mortality in infants,^11,12,13^ reduction of atopic disorders,^14,15,16^ prevention and treatment of type 1 diabetes,^17,18,19^ reduction in relapse of multiple sclerosis,^20^ and even reduction in COVID-19–associated morbidity and mortality in patients with diabetes.^21^ The most well-investigated nonspecific beneficial outcomes associated with BCG vaccine to date are those against cancers, including melanoma^22^ and bladder cancer,^23^ wherein intravesical delivery of BCG vaccine remains the highest standard treatment for non–muscle-invasive bladder cancer (NMIBC) to prevent tumor recurrence or progression.^24^

Some cohort studies^25,26,27^ have noted an intriguing and consistent association between the use of BCG vaccine for bladder cancer and a decreased incidence of ADRD. Limitations to these studies include sample size, cohort insularity, and/or analytical methods. Although historically, epidemiologic evidence has failed to successfully translate into clinical trial effectiveness,^28^ the promise of the affordability, safety, and accessibility of the BCG vaccine, its lack of use in most older adults, and its growing recognition as a clinically versatile and beneficial agent supports continued efforts to study its promise in preventing and treating ADRD. Herein, we examined the use of BCG vaccine treatment in patients with NMIBC and the risk of death and dementia. To our knowledge, we present the largest-to-date population health study of intravesical BCG vaccine for bladder cancer and ADRD, using electronic health records from patients affiliated with the Mass General Brigham (MGB) health care system. We applied a competing risks framework to address the issue that BCG vaccine treatment may prolong survival and thus put more individuals at risk for the development of dementia. We estimated time-invariant hazard ratios (HRs) using Cox proportional hazards regression and time-dependent cumulative incidence functions using a nonparametric approach.

## Methods

In this cohort study, we investigated whether intravesical BCG vaccine therapy for patients with NMIBC is associated with a decreased risk of developing ADRD. The MGB Institutional Review Board approved all work and waived the need for informed consent due to the low risk posed to patient privacy given the retrospective nature of the study. The Strengthening the Reporting of Observational Studies in Epidemiology (STROBE) reporting guideline was followed. Data were sourced from the MGB Research Patient Data Registry (RPDR), which includes structured and unstructured data sourced from MGB electronic health records. The study population comprised patients with NMIBC on initial cancer diagnosis. The intervention group was patients who received intravesical BCG vaccine therapy after NMIBC diagnosis. The control group was patients who did not receive any BCG vaccine therapy. The primary outcome was time from NMIBC diagnosis to ADRD onset.

### Inclusion and Exclusion Criteria

Patients aged 50 years or older with initial transurethral resection of bladder tumor (TURBT), a routine staging technique for bladder cancer, performed at MGB between May 28, 1987, and May 6, 2021, were included, with initial pathology results of NMIBC. Patients whose cancer progressed from NMIBC to muscle-invasive bladder cancer (defined as subsequent TURBT pathology report within 8 weeks showing MIBC) or radical cystectomy pathology report within 4 months of initial TURBT were excluded from analysis. Additional exclusions were a lack of at least 1 year of follow-up after the initial pathology report or a history or development of ADRD within 1 year of the initial pathology report.

### Participant Characteristics

In addition to age, sex and race and ethnicity were documented. Race and ethnicity categorization was based on self-reported values obtained from electronic health record profiles and was used to evaluate demographic characteristic differences between treatment groups.

### NMIBC Determination

The RPDR was queried for all surgical pathology reports. Natural language processing (NLP) was used to identify TURBT pathology reports, using the search term *bladder*, as well as other key words indicative of a TURBT, including *transurethral* and *cystoscopy*. The TURBT pathology reports were categorized as benign or malignant based on a combination of NLP using regular expressions (REGEX) and manual reviews. For reports of malignancy, cancer stage was determined by NLP with REGEX, categorizing patients as having NMIBC or MIBC according to standard TNM tumor classification. Natural language processing also was used to identify pathology reports suggesting prior cystectomy, locally invasive, or metastatic bladder cancer. Clinical stage from TURBT pathology reports was determined using a REGEX-based NLP algorithm developed by 2 practicing uro-oncologists (M.N. and A.Z.). Terms matching the REGEX results (eAppendix 1 in Supplement 1 for REGEX and pathology algorithm) were extracted from 100 random pathology reports. Ten iterative modifications were performed, with a final audit of 100 random pathology reports yielding 92% concordance of NLP-determined cancer stage with the American Urological Association guidelines.^29^ We defaulted to the 1973 World Health Organization 3-tiered pathology grading system^30^ given the incomplete transition to the 1998 standard across our data set.

### BCG Vaccine Treatment Determination

To identify patients with NMIBC who received BCG vaccine therapy, we used a combination of procedure codes, NLP, and manual validation by electronic health record review. Medical record contexts with the term *BCG* provided the initial data set. Manual validation was eased by highlighting terms that were highly specific for BCG vaccine treatment (eAppendix 2 in Supplement 1).

### ADRD Designation

Classification of ADRD was determined as previously described.^31^ This approach involves using dementia-related *International Classification of Diseases, Ninth Revision* (*ICD-9*) (290.X, 294.X, 331.X, and 780.93.X) or *International Statistical Classification of Diseases and Related Health Problems, Tenth Revision* (*ICD-10*) (G30.X, G31.X) codes and/or identification of drugs prescribed exclusively for ADRD (eg, galantamine, donepezil, rivastigmine, and memantine and their brand names).

### Comorbidity and Mortality Assessment

A Charlson Comorbidity Index score^32^ was generated for each person meeting inclusion criteria using electronic health record information before the date of the initial bladder pathology report. The Charlson Comorbidity Index score was computed using the R, version 4.2.1 comorbidity package (R Foundation for Statistical Computing)^33^ and the Quan *ICD-9* and *ICD-10* weighting schemes.^34^ The score was categorized as mild (0-2), moderate (3-4), severe (≥5), and missing. All-cause mortality information in the RPDR was sourced from electronic health records and the US Social Security Administration death master file, updated monthly.

### Statistical Analysis

Data analysis was performed from April 18, 2021, to March 28, 2023. We conducted inverse probability weighting (IPW) analyses to balance the 2 treatment arms with respect to sex, age, and Charlson Comorbidity Index score. We considered 2 competing time-to-event outcomes (time from initial NMIBC-determining pathology report to ADRD and time to death without ADRD) and assumed independent censoring. We conducted 3 types of analyses. The first of these was a Kaplan-Meier estimation of the survival curves and Cox proportional hazards regression for time to ADRD; these analyses aimed to replicate an evaluation performed by another group on a related data set.^25^ In the IPW Cox proportional hazards regression, *P* values correspond to the Wald test. Second, a competing risks IPW analysis that assumes the Cox proportional hazards regression structural models for the cause-specific hazard functions corresponding to 2 competing events was performed. Third, a competing risks IPW nonparametric analysis was done based on the weighted Aalen-Johansen estimator. In the second and third analyses, 95% CIs were obtained for each time point as 2.5% and 97.5% quantiles of sample distributions of bootstrap estimates. Analyses were run across the overall cohort and for 2 age strata (<70 and ≥70 years) based on a similar stratification scheme used in a related study.^26^ We focused on 2 treatment effect estimands: cause-specific HRs (both time-invariant HRs from the IPW Cox proportional hazards regression model and time-varying HRs from the IPW Aalen-Johansen estimators) and the risk differences, defined as the differences between the cumulative incidence functions (ie, risk functions^35^). The competing risks analysis was conducted using a previously developed causalCmprsk package in R.^36^ There were no missing data on age and sex; missingness for the Charlson Comorbidity Index score was added as a separate indicator variable. All analyses and plots were generated using R, version 4.2.1. Sensitivity analysis for residual confounding was estimated using E-values.^37,38^

## Results

### Participants and Descriptive Data

**Figure 1.**
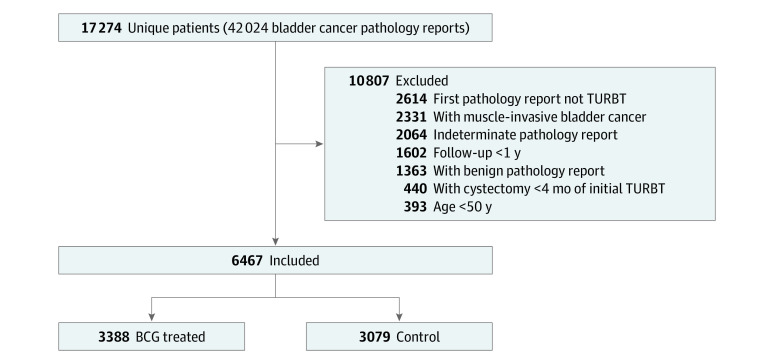
Patient Flowchart TURBT indicates transurethral resection of bladder tumor.

The RPDR query identified 42 024 bladder cancer pathology reports between 1987 and 2021. These reports represented 17 274 unique patients. Of these, 14 660 patients had a TURBT pathology report, 13 297 patients had an initial pathology report of malignancy, and 440 patients were excluded due to undergoing cystectomy within 4 months of the initial TURBT. After additional exclusion criteria were applied, 6467 patients were determined to have NMIBC. Of these, 3388 individuals were identified as having received BCG vaccine treatment and 3079 patients had not. (Figure 1). Demographic data comparing the BCG and control groups are presented in the Table. The overall mean (SD) age of the initial TURBT population was 70.3 (9.7) years. The BCG vaccine group comprised 783 women (23.1%) and 2605 men (76.9%) (mean [SD] age, 69.89 [9.28] years) vs the control group (903 [29.3%] women and 2176 [70.7%] men; mean [SD] age, 70.73 [10.0] years). In addition to the similarity in sex and age, the BCG vaccine and control groups were similar in race and ethnicity (standardized differences <0.1). The categories of the cancers differed (standardized difference <0.1) in that there were proportionately more BCG vaccine–treated patients with cT1 staging (more advanced, 33.4%) than in the control group (18.9%), with fewer patients in the BCG vaccine group in the cTa category (55.9%) than patients in the control group (73.3%). The BCG vaccine group overall showed more high-grade pathologic levels (53.4%) compared with the control group (24.5%). Charlson Comorbidity Index scores were similar between the groups, with most individuals classified as having mild comorbidities (62.4% overall).

**Table.** Demographic Characteristics

Characteristic	Participants, No. (%)		Standardized difference
	Control (n = 3079)	BCG vaccine (n = 3388)	
Age, mean (SD), y	70.73 (10.0)	69.89 (9.28)	−0.09
Sex			
Female	903 (29.3)	783 (23.1)	−0.06
Male	2176 (70.7)	2605 (76.9)	0.06
Race and ethnicity^a^			
African American or Black	55 (1.8)	50 (1.5)	0.00
American Indian	1 (0.03)	3 (0.08)	0.00
Asian	32 (1.0)	40 (1.2)	0.00
Hispanic or Latinx	31 (1.0)	21 (0.6)	0.00
Non-Hispanic White	2767 (89.9)	3136 (92.6)	0.03
Unknown	193 (6.3)	138 (4.1)	0.02
T category			
cTa	2257 (73.3)	1893 (55.9)	−0.17^b^
cTis	181 (5.9)	292 (8.6)	0.03
cT1	581 (18.9)	1130 (33.4)	0.14^b^
cT2	4 (0.1)	6 (0.2)	0.00
Indeterminate	56 (1.8)	67 (2.0)	0.00
Grade			
1 (Low)	1765 (57.3)	872 (25.7)	−0.32^b^
2 (Medium)	258 (8.4)	257 (7.6)	−0.01
3 (High)	753 (24.5)	1808 (53.4)	0.29^b^
Indeterminate	303 (9.8)	451 (13.3)	0.03
Charlson Comorbidity Index score			
0-2 (Mild)	1850 (60.1)	2184 (64.5)	0.04
3-4 (Moderate)	490 (15.9)	500 (14.8)	−0.01
≥ 5 (Severe)	315 (10.2)	245 (7.2)	−0.03
Missing	424 (13.8)	459 (13.5)	0.00
Follow-up time, mean (SD), y	7.00 (4.57)	6.79 (4.35)	0.045

^a^ Race and ethnicity were self-reported.

^b^ Absolute standardized difference greater than 0.1.

### Outcomes Data

#### BCG and Time to ADRD—Survival Analysis

**Figure 2.**
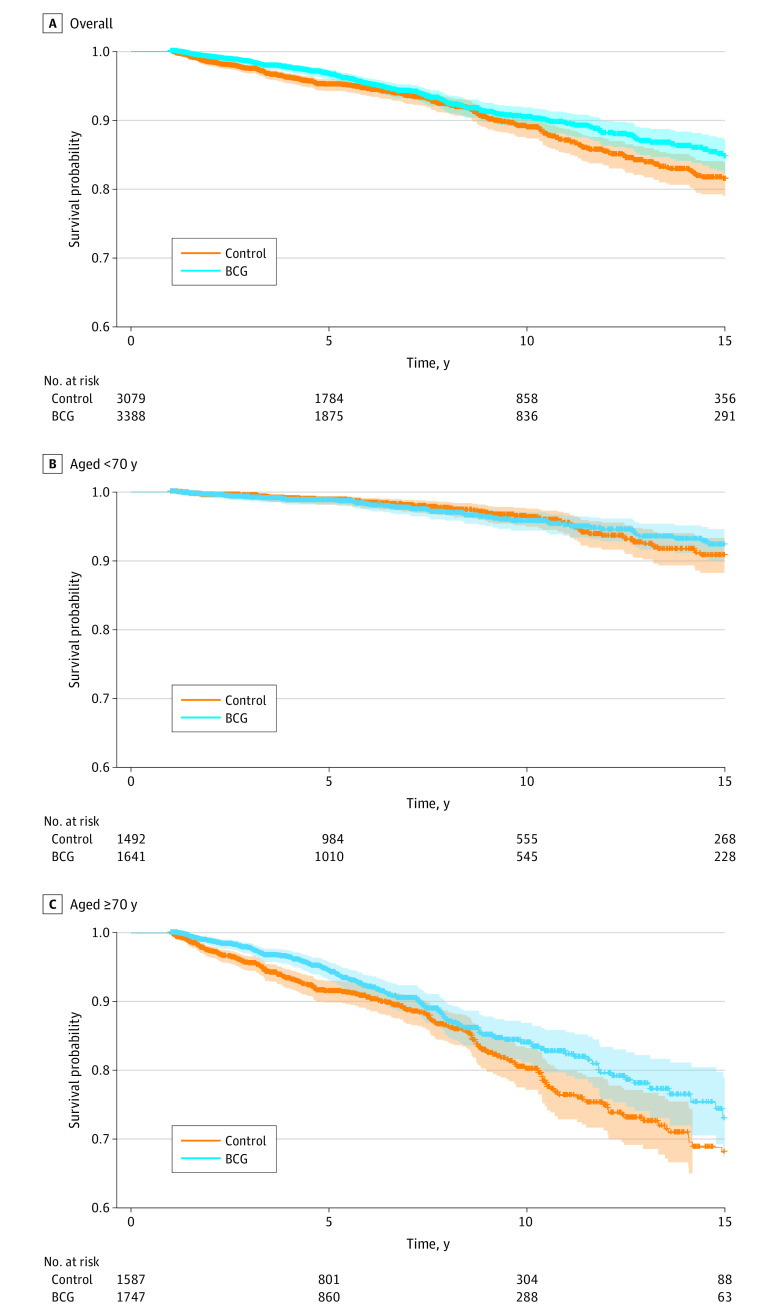
Overall and Stratified Alzheimer Disease and Related Dementia–Free Survival Survival, balanced for sex, age, and Charlson Comorbidity Index score. A, Overall cohort. B, Patients younger than 70 years. C, Patients aged 70 years or older.

To replicate a previous study that presumed death is an independent censoring event,^25^ we estimated the survival probability for the time-to-ADRD outcome using the Kaplan-Meier estimator and the HRs using IPW Cox proportional hazards regression (Figure 2). Treatment arms were balanced with respect to sex, age, race and ethnicity, and comorbidities using IPW. The overall number of individuals with an observed ADRD outcome during the follow-up period for the BCG vaccine group was 202, and in the control group, the number was 262. The ADRD incidence per 1000 person-years was 8.8 for the BCG vaccine and 12.1 for the control group. The population of patients younger than 70 years had similar incidence rates (4.3 per 1000 person-years for the BCG vaccine group and 4.8 per 1000 person-years for the control group), and patients aged 70 years or older had an incidence rate of 14.5 per 1000 person-years for the BCG vaccine group and 20.9 per 1000 person-years for the control group. We found a protective association of the BCG treatment with the time-to-ADRD outcome (N = 6467; HR, 0.80; 95% CI, 0.66-0.96; *P* = .02). In stratified analyses by age, the BCG vaccine treatment was associated with a lower risk of ADRD vs controls in patients aged 70 years or older compared with the overall cohort (n = 3334; HR, 0.74; 95% CI, 0.60-0.91; *P* = .005), whereas there was no association of treatment with time to ADRD in patients younger than 70 years (n = 3133; HR, 0.98; 95% CI, 0.68-1.40; *P* = .92) (Figure 2).

#### BCG Vaccine and Time to ADRD—Time to Death

**Figure 3.**
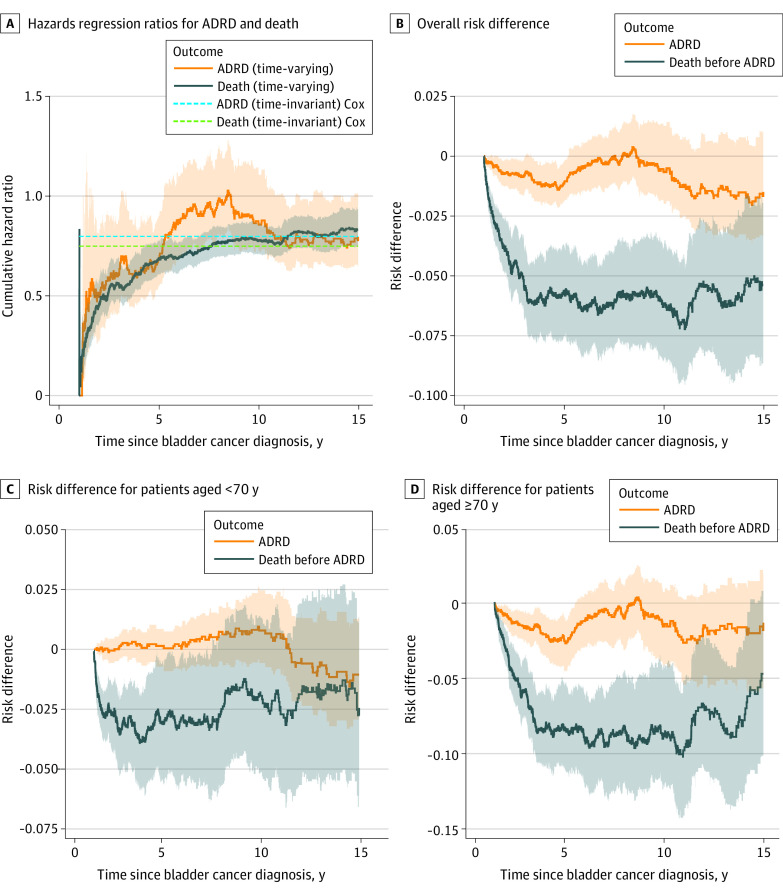
Competing Risks Analyses of Alzheimer Disease and Related Dementias (ADRD) and Death A, The time-invariant Cox proportional hazards regression ratios for ADRD (hazard ratio, 0.80; 95% CI, 0.69-0.99) and death (hazard ratio, 0.75; 95% CI, 0.69-0.82) and nonparametric (time-varying) cumulative hazard ratios based on weighted Aalen-Johansen estimators for ADRD and death; shaded areas of corresponding colors indicate 95% CIs. B, Overall risk difference for ADRD and death before ADRD; shaded areas of corresponding colors indicate 95% CIs. C, Stratified analyses of patients younger than 70 years; shaded areas of corresponding colors indicate 95% CIs. D, Stratified analyses of patients aged 70 years or older; shaded areas of corresponding colors indicate 95% CIs.

The total number of deaths in the BCG vaccine group was 751 and, in the control group, 973. The subcohort of patients younger than 70 years had 300 deaths in the BCG vaccine group and 311 in the control group; in patients aged 70 years or older, there were 451 deaths in the BCG vaccine group and 662 in the control group. Since death is a competing event that might preclude the occurrence of ADRD, we performed a competing risks analysis using IPW to adjust for potential confounders. We estimated time-invariant cause-specific HRs from the Cox proportional hazards regression model and the time-varying cause-specific HRs using the nonparametric weighted Aalen-Johansen estimators for both competing events. We noted an association suggesting protection between the BCG vaccine and ADRD, as well as between the BCG vaccine and competing risk of death (Figure 3A). In a cause-specific Cox proportional hazards regression model with death as a competing risk and IPW for emulation of baseline randomization, the estimated cause-specific HR for ADRD was 0.80 (95% CI, 0.69-0.99) and for death was 0.75 (95% CI, 0.629-0.82) for patients treated with the BCG vaccine compared with controls. In the time-varying nonparametric analysis, we estimated the risk differences between the BCG vaccine and control arms (defined as the differences between the cumulative incidence functions for both ADRD and death without ADRD). The 5-year risk difference for ADRD was −0.011 (95% CI, −0.019 to −0.003) and for mortality was −0.056 (95% CI, −0.075 to −0.037). After 5 years, the risk difference for ADRD was not statistically significant, as the 95% CIs included the null effect mark. However, the risk of ADRD was still lower in the BCG vaccine–treated group throughout the whole follow-up period (Figure 3B). In stratified analyses, a 5-year risk difference of −0.021 (95% CI, −0.038 to −0.004) for ADRD and −0.083 (95% CI, −0.109 to −0.057) for mortality was observed in patients aged 70 years or older (Figure 3C), but no risk difference was noted for ADRD (0.001; 95% CI, −0.009 to 0.008), and a risk difference of −0.026 (95% CI, −0.051 to −0.001) was observed for mortality in those younger than 70 years (Figure 3D).

## Discussion

### BCG Vaccine and Risk of ADRD

We observed an association between the use of BCG vaccine treatment suggesting usefulness for patients with NMIBC and who are at risk for ADRD. Competing risks analysis reiterates this inverse association despite a pronounced reduction in mortality associated with treatment. Thus, patients appear to live longer and have a reduced incidence of ADRD after BCG vaccine treatment. The results of this study, which is, to our knowledge, the largest to date are consistent with findings from other groups^25,26,27^ suggesting ADRD protection as an additional nonspecific use of this pleiotropic biologic agent. This finding—like a recent report in which BCG vaccine for patients with type 1 diabetes also led to decreased COVID-19–related morbidities^21^—suggests a secondary association of BCG with a lower incidence of ADRD and mortality, in which the primary use of the BCG vaccine was to treat bladder cancer vs its standard use in tuberculosis.

Using nonparametric analysis, we found that, although the results were not statistically significant after 5 years, the probability of incident ADRD was lower in the BCG vaccine–treated group despite lower mortality rates throughout the follow-up period. This lower probability was more pronounced in the subcohort of patients who were diagnosed with NMIBC and started BCG vaccine treatment when aged 70 years or older; there was no association in the younger subcohort. Several factors may explain these time-dependent findings. First, fewer individuals with data available after a 5- or 10-year follow-up leads to wider 95% CIs. Second, with time, patients were more likely to be lost to follow-up or die. There was not a decreased risk difference of ADRD vs mortality over time but rather an increase in uncertainty and an overall greater mean risk difference. Alternatively, there may be a lower risk of ADRD associated with earlier BCG vaccine treatment; the initiation and maintenance schedule of BCG vaccine treatment of bladder cancer is individualized, but treatments are more frequent nearer to the initial NMIBC diagnosis.

A recent cohort study observed a dose-based protective association of BCG with the incidence of ADRD.^27^ We were unable to measure dosing in our cohort accurately; the MGB RPDR consists of data from a mixed population of individuals who received BCG vaccine treatment in the MGB health care system and elsewhere. In addition, detailed data on patients treated by MGB hospital urology groups during earlier years with this cohort may have been captured only in paper medical records inaccessible at the present time and not in the hospital-based electronic health records. Furthermore, as a tertiary care center, in individuals with scant bladder cancer history in their electronic health records, earlier BCG vaccine treatment is described but not quantified in notes.

### Mechanism of BCG Vaccine in ADRD

Our understanding of the mechanisms associated with the nonspecific medicinal value of the BCG vaccine come largely from experimental infectious disease–related studies.^39,40^ The BCG vaccine induces epigenetic changes to the innate immune system,^39,40,41^ resulting in a long-lasting, more robust response to heterogeneous pathogens (ie, trained immunity).^42^ The BCG vaccine has also shown benefits in lowering hemoglobin A_1c_ in individuals with type 1 diabetes^17,18,19^ and in reducing magnetic resonance imaging evidence of multiple sclerosis–related lesions.^20,43,44^ Notably, the diabetes-related benefits of the BCG vaccine can take years to appreciate^17^ and are associated with epigenetic changes in T-regulatory cells.^45^ Maintenance treatment with BCG vaccine for NMIBC similarly entails multiple treatments a year for 3 years, increasing the robustness of the host immune response and decreasing the likelihood of recurrence in a dose-dependent manner.^46^ This prime boost mechanism^47^ shares the conceptual framework of trained immunity from infectious disease. Immune retraining or tolerance induction through peripheral inflammatory stimuli can exacerbate or mitigate Alzheimer disease pathologic factors.^48^ Preclinical studies of BCG vaccine in an Alzheimer disease mouse model found reduced cognitive deficits accompanied by markedly high CD45, interleukin-10–secreting monocyte infiltration into choroid plexus and perivascular spaces along with anti-inflammatory shifts in brain-derived cytokine levels and increased brain-derived neurotrophic factor,^49^ greater neuronal dendritic complexity, and higher postsynaptic density-related protein levels.^50^

### BCG Vaccine and Increased Survival

Although largely similar to the control group, the BCG vaccine group had an overall higher grade of malignancy and higher stage of disease. The BCG vaccine is generally recommended for patients having intermediate and high-risk bladder cancer^29^; thus, most patients receiving BCG vaccine have high-grade pathologic levels. Other reasons for not receiving BCG vaccine include immunocompromised status and active tuberculosis infection. Based on the overall higher risk of disease progression in the BCG vaccine–treated group, it might be anticipated that the BCG vaccine group, having a more aggressive cancer, would have a higher mortality rate, masking the incidence of ADRD. A sensitivity analysis removing patients who developed metastatic cancer did not seem to affect the associated mortality benefit of BCG vaccine.

### Limitations

This study has several limitations. First, *ICD-9* and *ICD-10* diagnostic codes and medications underestimate the incidence of ADRD.^51^ Second, we presumed time-linked treatment with NMIBC diagnosis and could not accurately capture BCG dosing. Third, residual confounding may account for the association between BCG vaccine and ADRD and mortality. For instance, frailty and dementia onset and ADRD biomarkers are closely linked,^52^ and frailty, while not a contraindication for BCG vaccine treatment, may increase treatment intolerability. Sensitivity analyses suggest that a confounder associated with ADRD and BCG treatment (eg, frailty) with a relative risk of at least 1.11 could negate the observed BCG vaccine association with the time to ADRD.^37^A confounder with a relative risk of at least 1.44 would be necessary for the observed BCG association with death to become nonsignificant.

## Conclusions

In this cohort study of individuals diagnosed with NMIBC, we identified an association between BCG vaccine treatment and reduced risk of ADRD and all-cause mortality, although the risk differences varied with time. Well-designed interventional trials will be helpful in further studying this pleiotropic vaccine in the context of ADRD.

## Notes

Supplement 1. **eAppendix 1.** Bladder Cancer (BLC) Pathology REGEX Terms and Algorithm **eAppendix 2.** REGEX Identifiers of BCG Terms Highly Likely to Represent Recent or Prior BCG Treatment

Supplement 2.
Data Sharing Statement
