# Supplementary material for: Association of BCG Vaccine Treatment With Death and Dementia in Patients With Non–Muscle-Invasive Bladder Cancer
Source: JAMA Netw Open. 2023 May 19;6(5):e2314336. doi: 10.1001/jamanetworkopen.2023.14336 (PMC10199345; doi:10.1001/jamanetworkopen.2023.14336)
Supplement: Supplement 1. — eAppendix 1. Bladder Cancer (BLC) Pathology REGEX Terms and Algorithm eAppendix 2. REGEX Identifiers of BCG Terms Highly Likely to Represent Recent or Prior BCG Treatment [file jamanetwopen-e2314336-s001.pdf]

Supplementary Online Content

Weinberg MS, Zafar A, Magdamo C, et al. Association of BCG vaccine treatment with death and dementia in patients with non–muscle-invasive bladder cancer. *JAMA Netw Open*. 2023;6(5):e2314336. doi:10.1001/jamanetworkopen.2023.14336

eAppendix 1. Bladder Cancer (BLC) Pathology REGEX Terms and Algorithm

eAppendix 2. REGEX Identifiers of BCG Terms Highly Likely to Represent Recent or Prior BCG Treatment

This supplementary material has been provided by the authors to give readers additional information about their work.

© 2023 Weinberg MS et al. *JAMA Network Open.*

e<u>Appendix 1</u>: *Bladder cancer (BLC) pathology REGEX terms and algorithm*
***(x): *** = any character, x = maximum number of characters or words permitted (specified)

Bladder-specific pathology report (negating terms for pathology reports)

- status post cystectomy
- brain biopsy
- specimen type: liver
- specimen type: stones
- molecular test for solid fusion assay
- part : ileal conduit
- colon biopsy, rectum
- specimen type: bone marrow

BLC dividing into TURBT or Cystectomy

- Bladder_reporttype = TURBT if contains
    - transurethral or trans-urethral & resection
    - bladder biops
    - bladder bx
    - deep biops
    - wall), biops
    - wall, biops
    - bladder (cold cup
    - bladder, biops
    - bladder (Dome Lesion), Biopsy
    - random bladder
    - bladder (tumor), biopsy
    - bladder tumor
    - cystoscopy
    - TURBT
    - bladder ***(15 char) biopsy
    - biopsy*(1 word)
    - WITHOUT prostate, transurethral OR prostate, trans-urethral OR transurethral resection ***(1 word) prostate, OR trans-urethral resection ***(1 word) prostate
    - WITHOUT prostate (T.u.r.) or TUR prostate
    - bladder ***(1 word) biopsy
    - did not find term urinary bladder biopsy, cystoscopy, TURBT
    - “bladder (dome), biopsy” not found
    - bladder ***(25 char) TUR
    - bladder, transurethral resection
    - bladder transurethral resection
- Bladder_reporttype = cystectomy if contains
    - cystectomy
    - cystoprostatectomy
    - Lymph node
    - Bladder ***(10 char) excision

© 2023 Weinberg MS et al. *JAMA Network Open.*

    - WITHOUT gall OR gallbladder in operation field

TURBT specific

- T Stage
    - tstage = cTis if CIS = & not cTa or cT1-4
    - tstage = cTX if contains
    - tstage = cTa if contains
        - cTa
        - (Ta)
        - , Ta.
        - no invasion into lamina propria is identified
        - no invasion of lamina propria is identified
        - carcinoma. no invasion is identified
        - no lamina propria or lymphatic vascular invasion is identified
        - note). no invasion is identified.
        - grade non-invasive
        - grade, non-invasive
        - grade. no invasion is present
        - grade. no invasion identified.
        - grade, with no lamina propria
        - Non-invasive papillary urothelial carcinoma
        - 3, non-invasive
        - 3, NONINVASIVE
        - 3), non-invasive
        - 3). No invasion
        - 3). No lamina propria invasion.
        - 3 (non-invasive
        - 3, invasion not demonstrated
        - 3. No lamina propria invasion
        - 3. No invasion present.
        - No invasive carcinoma
        - No invasion of lamina propria
        - Did not find this term: noninvasive (case-sensitive)
        - No ***(15 char) lamina propria
        - No invasion demonstrated
        - No invasive
        - Non-invasive
        - Noninvasive
        - No lamina propria is present
        - No submucosa
        - Invasion is not seen
        - No ***(15) lamina propria
        - Not definite for lamina propria
        - Without invasion
        - Without definite invasion
        - Invasion of lamina propria is not

© 2023 Weinberg MS et al. *JAMA Network Open.*

    - Invasion of lamina propria cannot
    - No lamina invasion
- tstage = cT1 if contains
    - T1
    - invades lamina propria
    - invades the lamina propria
    - invasive into the lamina propria
    - present as lamina propria invasion
    - invasive at least into lamina propria
    - superficial invasion into lamina propria
    - superficially invasive high grade
    - superficially invasive low grade
    - invasive ***(15 char) urothelial carcinoma
    - invasive ***(20 char) carcinoma
    - *(1 char)carcinoma ***(1 word) widely invasive
    - With invasion of lamina propria
    - superficial invasion of lamina propria
    - focal invasion of lamina propria
    - microscopic invasion of lamina propria
    - suspicious for invasion of lamina propria
    - invading lamina propria
- tstage = cT2 if contains
    - T2
    - invades the muscularis propria
    - invading the muscularis propria
    - invades into the muscularis propria
    - invades muscularis propria
    - invasive into muscularis propria
    - with muscle invasion
    - extension into muscularis propria
        - invad***(20) muscularis propria ("not" cannot appear 5 characters prior)
    - extension into the muscularis propria is present
    - extension into the muscularis propria is present
    - invasive of the muscularis propria
    - tumor invade the muscularis propria
    - invading into muscularis propria
    - invading muscularis
    - invasive of muscularis
    - suspicious for muscularis propria invasion
    - invasion into muscularis propria is present
- Tstage = cT3 if contains
    - T3
    - invasive through the muscularis propria into the perivesical fat
- Tstage = cT4 if contains
    - rectal mucosa with ***(12) invasion of carcinoma

© 2023 Weinberg MS et al. *JAMA Network Open.*

        - rectal mucosa ***(25) with invasion of carcinoma
    - Create new variable maximum_tstage based on maximum if multiple stages described, or only stage if solitary description of stage reported
- Muscle in specimen
    - Musclepresent = 1 if tstage=(cT2 or cT3) OR contains:
        - muscularis propria is present
            - Should not be preceded by 'no' (see below)
        - muscularis propria present
            - Should not be preceded by 'no' (see below)
        - muscularis propria) is present
        - muscularis propria is focally present
        - negative muscularis propria
        - fragment of muscularis propria uninvolved by tumor
        - benign urothelial mucosa and muscularis propria
        - muscularis propria are present
    - Musclepresent = 0 not musclepresent = 1 & contains
        - propria is not present
        - propria not identified
        - no muscularis present
        - no muscularis seen
        - no muscularis propria present
        - no muscularis propria is identified
        - no muscularis propria is present
        - . no muscularis propria.
        - recognizable muscularis propria cannot be identified

Cystectomy specific

- T Stage
    - cT2 if contains
        - cT2
    - cT3 if contains
        - invasive through the bladder wall and into adjacent adipose tissue
        - cT3
        - into the perivesicular fat
- N stage
    - N0 if contains
        - N0
    - N1 if contains
        - N1
- total lymph nodes
- positive lymph nodes
- margins
    - all margins are free of invasive carcinoma

Other non-NMIBC

- no tissue present
- fine needle aspirate
- prostate*(1 char)

© 2023 Weinberg MS et al. *JAMA Network Open.*

- prostate, TUR
- transurethral prostate
- S99N49578 (possible carcinoma but inconclusive)

© 2023 Weinberg MS et al. *JAMA Network Open.*

eAppendix 2: *REGEX Identifiers of BCG terms highly likely to represent recent or prior BCG treatment*

\badministration\s*visit\b
\bampule\b
\bdiluent\b
\benters\s*the\s*urology\s*office\b
\blive\b
\breconstituted\b
\bsaline\b
\bsterile\b
\bstrength\b
\bthera\-cys\b
\bthera\s*cys\b
\btheracys\b
\btice\b
\btreatment\s*\#\B
\btreatment\s*week\b
\bvial\b
1\s*of\s*6|2\s*of\s*6|3\s*of\s*6|4\s*of\s*6|5\s*of\s*6|6\s*of\s*6
\bdose\s*\#\B
1_\s*of\s*6|2_\s*of\s*6|3_\s*of\s*6|4_\s*of\s*6|5_\s*of\s*6|6_\s*of\s*6
\bbcg\s*instillation\s*was\s*administered\b
\breceived\b.{0,30}\bbcg\b
\bstarted\b.{0,30}\bbcg\b|\bbcg\b.{0,30}\bstarted\b
\btreated\s*with\b.{0,20}\bbcg\b
\bundergoing\b.{0,20}\bbcg\b
\binstilled\b.{0,10}\bbcg\b|\bbcg\b.{0,10}\binstilled\b
\bx6\b.{0,20}\bbcg\b|\bbcg\b.{0,20}\bx6\b
\bx3\b.{0,20}\bbcg\b|\bbcg\b.{0,20}\bx3\b
\B\#\B.{0,10}\bbcg\b|\bbcg\b.{0,10}\B\#\B
\bhad\b.{0,10}\bbcg\b|\bbcg\b.{0,10}\bhad\b
\b6\s*weeks\b.{0,10}\bbcg\b|\bbcg\b.{0,10}\b6\s*weeks\b
\b6\s*wk\b.{0,10}\bbcg\b|\bbcg\b.{0,10}\b6\s*wk\b
\bhere\s*for\b.{0,10}\bbcg\b
\bhere\s*to\b.{0,10}\bbcg\b
\bpresent\s*for\b.{0,10}\bbcg\b
\bs\/p(?!.*\bTURBT\b).{0,15}\bbcg\b
\b6\b.{0,10}\bbcg\b|\bbcg\b.{0,10}\b6\b
\bstatus\s*post(?!.*\bTURBT\b).{0,15}\bbcg\b
\bcycles\b.{0,10}\bbcg\b|\bbcg\b.{0,10}\bcycles\b
\bTURBT\b.{0,15}\bbcg\b
\bx[1-6]\b.{0,10}\bbcg\b|\bbcg\b.{0,10}\bx[1-6]\b
\bunderwent\b.{0,20}\bbcg\b
\binstillations\b.{0,10}\bbcg\b|\bbcg\b.{0,10}\binstillations\b
\b6\s*week\b.{0,10}\bbcg\b|\bbcg\b.{0,10}\b6\s*week\b

© 2023 Weinberg MS et al. *JAMA Network Open.*
