# Supplementary material for: Association of BCG Vaccine Treatment With Death and Dementia in Patients With Non–Muscle-Invasive Bladder Cancer
Source: JAMA Netw Open. 2023 May 19;6(5):e2314336. doi: 10.1001/jamanetworkopen.2023.14336 (PMC10199345; doi:10.1001/jamanetworkopen.2023.14336)
Supplement: Supplement 2. — Data Sharing Statement [file jamanetwopen-e2314336-s002.pdf]

Data Sharing Statement

Weinberg. Association of BCG Vaccine Treatment With Death and Dementia in Patients With Non–Muscle-Invasive Bladder Cancer. *JAMA Netw Open*. Published May 19, 2023. doi:10.1001/jamanetworkopen.2023.14336

Data
Data available: Yes
Data types: Deidentified participant data
How to access data: Researchers can obtain an anonymized version of the study dataset from the authors upon request and completion of the MGB Health data use agreement. Please email das5@mgh.harvard.edu with requests. For RPDR data visit: http://rpdr.partners.org/ or https://rc.partners.org/research-apps-services/identify-subjects-request-data#getstarted
When available: With publication

Supporting Documents
Document types: Statistical/analytic code
How to access documents: See supplemental materials for REGEX NLP
When available: With publication

Additional Information
Who can access the data: Researchers whose proposed use of the data has been approved
Types of analyses: Per MGB Health data use agreement
Mechanisms of data availability: Researchers can obtain an anonymized version of the study dataset from the authors upon request and completion of the MGB Health data use agreement.
